# An unusual case of a large fibroepithelial stromal polyp presenting as a nipple mass

**DOI:** 10.1186/1756-0500-6-345

**Published:** 2013-08-30

**Authors:** Abeer M Shaaban, EPL Turton, William Merchant

**Affiliations:** 1 Department of Histopathology and Molecular Pathology, St James’s University Hospital, Level 5 Bexley Wing, Leeds LS9 7TF, UK; 2 Department of Breast and Endocrine Surgery, St James’s Hospital, Leeds, UK

**Keywords:** Breast, Nipple, Fibroepithelial polyp

## Abstract

**Background:**

Fibroepithelial stromal polyps (FESP) are benign lesions that typically occur in the genital area and are known to represent a diagnostic challenge for pathologists. Not only do they have a spectrum of morphological changes that ranges from bland morphology to rather atypical appearances, but they also share morphological features with a number of benign and malignant lesions.

This is a report of a rare presentation of a FESP of the breast.

**Case presentation:**

We describe an unusual case of a large polypoid mass arising from the nipple and connected to it by a long pedicle in a female of 45. The lesion comprised spindle and stellate shaped cells with bizarre stromal giant cells. The morphological and immunohistochemical diagnostic features are provided together with a discussion of possible mimics.

**Conclusion:**

FESPs may occur in the female breast. It is important to differentiate the lesion from other benign and malignant spindle cell lesions particularly metaplastic carcinoma.

## Background

Fibroepithelial stromal polyps (FESP) are benign lesions recognised in the skin, oral cavity, urinary tract [1] and genital area [2]. They are recognised as posing diagnostic problems in the vulvo-vaginal region for the practising pathologist. These lesions, apart from being uncommon, have overlapping morphological features with other mesenchymal lesions specific to the vulvovaginal area. These lesions include angiomyofibroblastoma, aggressive angiomyxoma, and cellular angiofibroma. Other lesions that can morphologically mimic FESP are leiomyomas, superficial angiomyxoma, perineureomas and neurofibromas. Although immunohistochemistry has traditionally been described to assist differentiating these lesions, they are of limited clinical value.

FESP are remarkable for their ability to exhibit a wide range of histological appearances. They present in young or middle aged women most commonly in the vagina but also in the vulva [3] and rarely the cervix [4].

Only the clinical presentation of one case of FESP has been recently described in the breast [5]. Here, we present a case of a fibroepithelial stromal polyp arising in the nipple and showing histological appearances of those described in the vulvovaginal region.

## Case presentation

**Figure 1:**
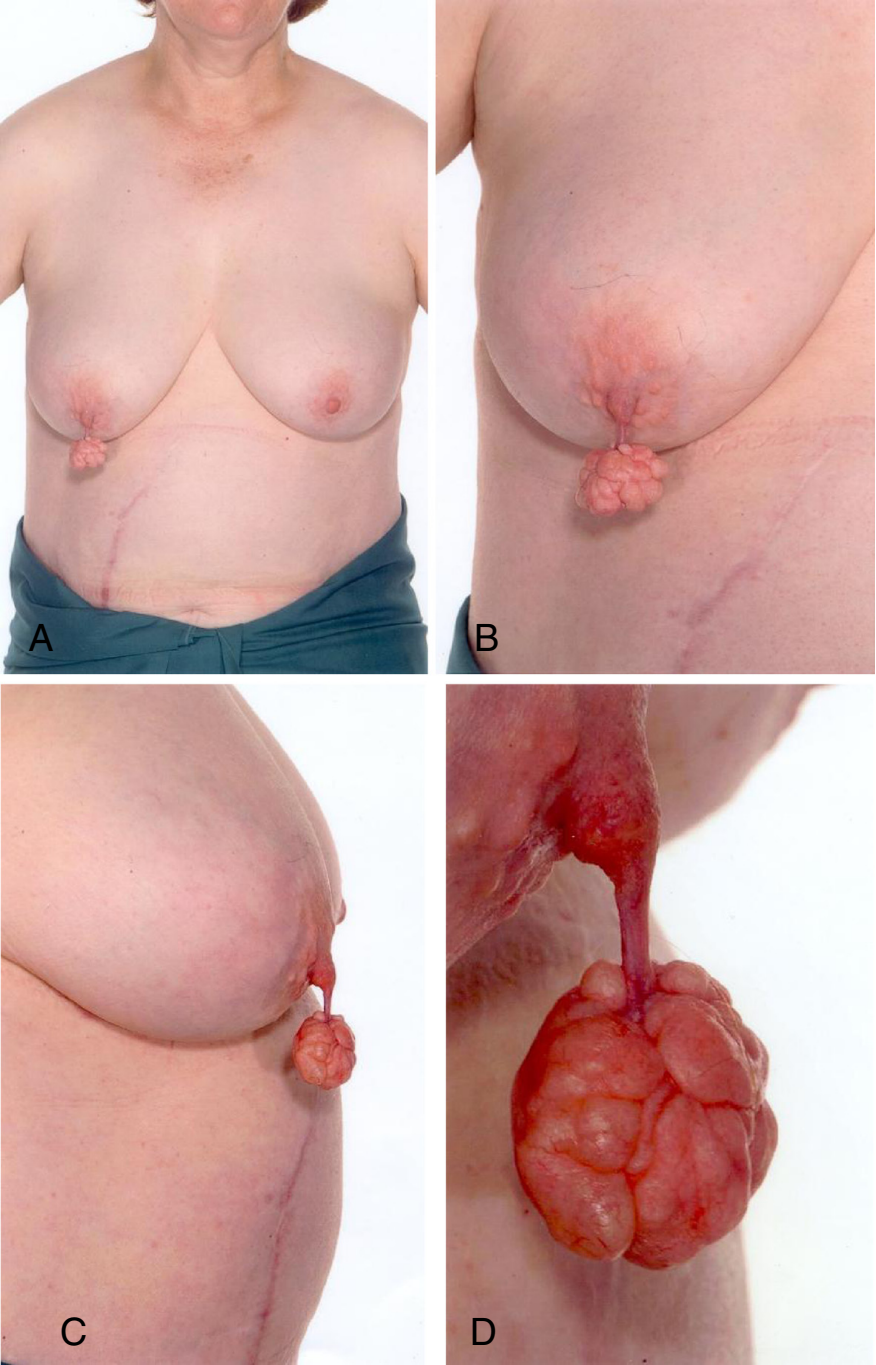
**Clinical presentation of the lesion. (A-D)**: a polypoid mass originating from the nipple, to which it is connected by a long pedicle. Both breasts are otherwise unremarkable.

This is a 45 year-old lady with no relevant medical history. She presented with a 4x4cm painless nipple lump (Figure 1). The lump has been present for a long time and slowly increased in size. The patient was not pregnant and the increase in size was not related to a previous pregnancy. On clinical examination, the mass was well circumscribed, firm and covered by a stretched but otherwise unremarkable skin. It was connected to the nipple by a long pedicle (Figure 1A-D). The rest of the breast was normal. Breast imaging was otherwise unremarkable.

Clinically, the lesion was likely to be benign though rather unusual. The clinical differential diagnosis included a possible leiomyoma or dermatofibroma.

The patient was treated by local excision of the lesion under general anaesthetic as a day case procedure with no post-operative complications. No further local treatment was given.

The specimen was examined by both breast and soft tissue pathologists by H&E and immunohistochemistry. The latter included EMA, desmin, calponin, caldesmon, EMA, S100, CD34, factor XIIIA, oestrogen receptor and progesterone receptor.

### Macroscopically

The lesion was a well-defined, polypoid mass that exhibited a pedicle. The mass was firm with a uniform, translucent, whorly, greyish cut section. Apart from the base of the pedicle, the lesion was covered by unremarkable skin. There was no evidence of haemorrhage or necrosis.

### Microscopically

**Figure 2:**
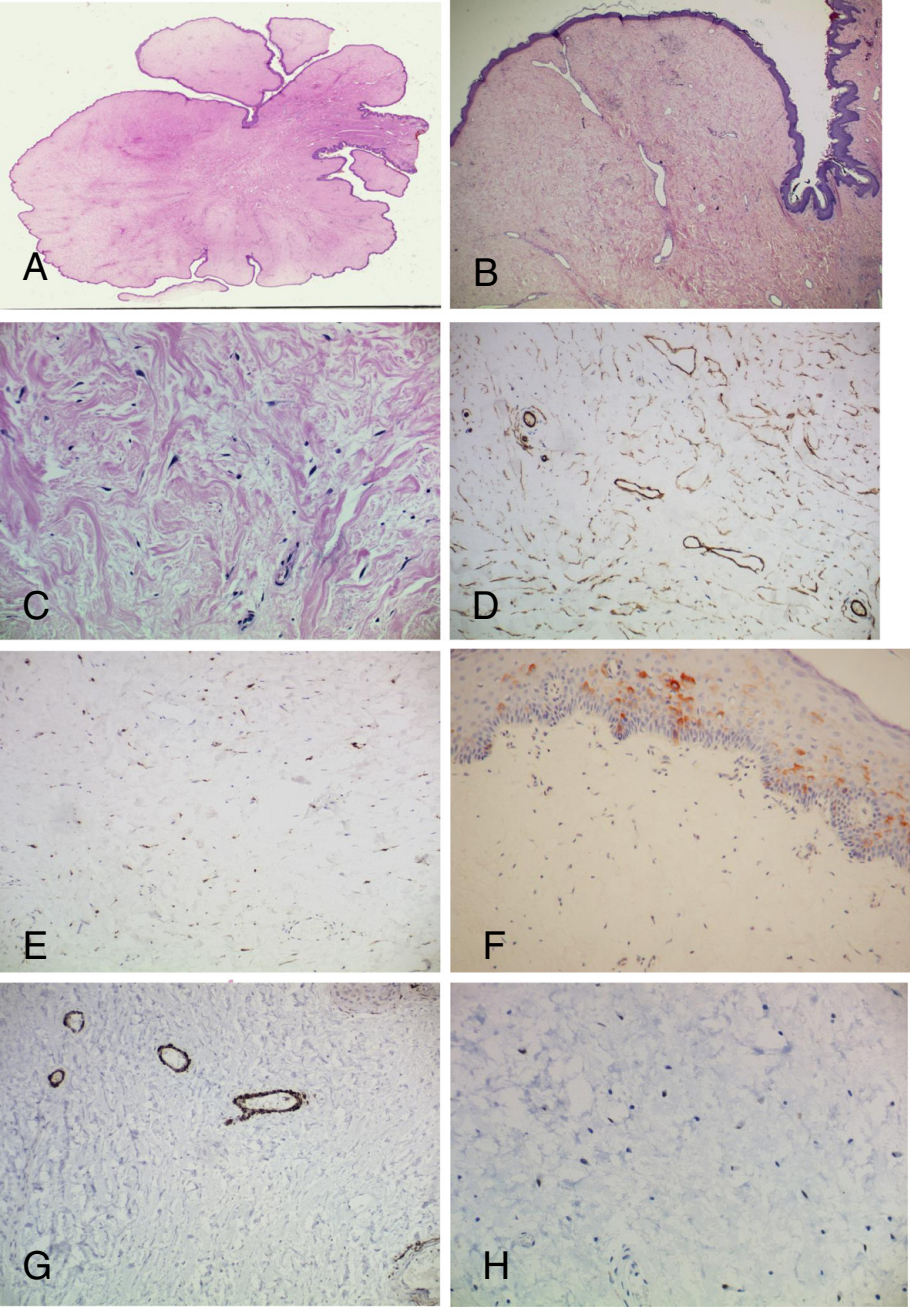
**Histological appearances of the lesion. A**. Low power view of the lesion illustrating the polypoid appearance (x20). **B** and **C**: The lesion is rather hypocellular. The stroma comprised spindle and stellate shaped cells B(x100) and C(x400). **D**- CD34 immunohistochemistry showing a large number of variably-sized blood vessels within the stroma (x200). **E**- Stromal cells are positive for factor X111a (x400). **F**- The cells are negative for EMA. Note positivity of the overlying epithelium (x200). **G**- Caldesmon showing positive expression in the muscle coat of the lesional vessels. Stromal cells are not stained (x200). **H**- Stromal cells are negative for oestrogen receptor (x400).

Sections revealed a rather hypocellular lesion that extended upwards to the overlying epidermis (Figure 2A-C). There was no demarcation between the lesion and the epidermis. The stalk comprised fibromuscular tissue of the nipple and was covered by unremarkable skin including sebaceous units. The lesion comprised a haphazard proliferation of rather bland spindle and stellate-shaped cells. The lesion showed variable cellularity with markedly hypocellular stroma alternating with more cellular areas. Occasional multinucleate stromal giant cells were seen. A sprinkle of mast cells was seen within the stroma. Scattered thin walled blood vessels were seen. There was no evidence of necrosis and mitoses were not a feature. No plasmacytoid cells, thick-walled vessels or cells with epithelioid morphology were seen.

Immunohistochemistry showed the lesional cells to be focally immunoreactive for smooth muscle actin (5% of cells were stained), and CD34 but negative for desmin, calponin, caldesmon, EMA, S100, oestrogen receptor and progesterone receptor (Figure 2D-H). Scattered through the lesion were factor XIIIA positive dendritic-like cells (approximately 45% of cells were positive).

The patient remained well and follow-up showed no regrowth or recurrence.

## Discussion

In this report we present a case of 45 year old female presenting with a large polypoid nipple mass. The site and macroscopic appearances were rather unusual. The case was diagnosed as a benign fibroepithelial stromal polyp reminiscent of the lesions described in the female genital area. Only one recently published report described a similar clinical presentation in a 35 year old female [5]. However from the brief histological description provided, the lesion does not appear to contain stellate or giant stromal cells and therefore may not be identical to the current case.

Histologically, the lesions are characteristically polypoid and usually contain a conspicuous fibrovascular core. The stroma is the most distinctive aspect of the lesion and can exhibit a wide range of appearances. The stroma can be hypocellular, being composed of bland spindle shaped cells with indistinct cytoplasm set within a loose or finely collagenous matrix. Stellate and multinucleate stromal cells are characteristic, being more noticeable at the epithelial-stromal interface [6]. At the other end of the spectrum, the stroma of some FESP exhibit marked cellularity, nuclear pleomorphism and increased mitotic activity including atypical mitoses. These lesions were called “cellular pseudoangiomatous fibroepithelial stromal polyps” [6]. These lesions are morphologically worrisome and may be mistaken for a malignant neoplasm. Cellular FESP tend to exhibit a greater degree of cellularity in the centre of the lesion, becoming less cellular as the lesion extends up to the stromal epithelial interface [6]. The stellate giant cells in FESP of the oral cavity are shown to be derived from fibroblastic lineage [7].

Other lesions that occur in the breast have also been reported in the genital area (e.g. ectopic breast tissue, fibroadenomas, mammary carcinomas) [8,9]. The mammary line extends from the breast superiorly to the genital area and therefore, it is conceivable that certain lesions can be shared between both areas.

The morphological features of the reported lesion are fully in keeping with a benign fibroepithelial stromal polyp. This polypoid lesion abuts the overlying epidermis without a distinct Grenz zone. It had the stellate cells and occasional bizarre multinucleate cells characteristic of FESPs. A conspicuous fibrovascular core was identified together with a sprinkle of mast cells. There were no features to suggest malignancy and no mitoses were seen in the lesion. The MIB-1 proliferation marker is of limited diagnostic value in those lesions and there is no established cut off value for positivity to help separate from other mimics.

FESPs show variable morphological appearances and, as in the vulvovaginal area, need to be distinguished from other mesenchymal lesions. In the breast, a wide range of benign and malignant spindle cell lesions occur in the breast; for review see [10]. In this context, it is important to differentiate spindle cell metaplastic carcinoma-which can appear deceptively bland, from other mesenchymal lesions [11]. This current case shows no expression of epithelial markers. Atypical neurofibroma and pleomorphic fibroma are possible differentials. The former is S100 positive (negative in the current case). Pleomorphic fibroma has atypical single fibroblasts which are CD34 positive and giant cells are not a feature. In the current case, atypia was limited to giant cells and CD34 was only focally positive. Given the postulated origin, the FESPs should also be differentiated from the entity: psudosarcoma botryoides (fibroepithelial polyps with atypical stromal cells) that was previously described in the vulva and vagina. Although histologically benign, two out of the 13 case series described recurred after incomplete excision [3]. Sarcoma botryoides (embryonal rhabdommyosarcoma), on the other hand, is a frankly malignant tumour that arises under the mucosal surfaces of body orifices such as vagina, bladder and cervix [12]. They often occur at a younger age (childhood, adolescence). The characteristic cambium layer and the pleomorphic spindle cells with rhabdomyoblasts were not seen in the presented case.

The significance of the focal staining with factor XIIIA and CD34 is uncertain. Factor XIIIA stains dermal dendrocytes and is expressed in a variety of mesenchymal skin tumours e.g. dermatofibromas and fibrous papule of the face. CD34 also stains a wide variety of mesenchymal tumours but the focal nature of expression in this case would reduce any diagnostic significance.

## Conclusions

FESPs can occur in the female breast. The presentation is of a long standing slowly growing pedunculated polyp, arising from the nipple. The histological features are those of a variably cellular lesion with spindle, stellate and stromal giant cells. The cells are focally positive for smooth muscle actin but negative for caldesmon, calponin and epithelial markers. In the breast, it is important to differentiate the lesion from metaplastic (spindle cell) carcinoma which can present as a relatively bland spindle cell proliferation.

## Consent

Written informed consent was obtained from the patient for publication of this case report and any accompanying images. A copy of the written consent is available for review by the Editor of this journal.

## Abbreviations

FESP: Fibroepithelial stromal polyp; EMA: Epithelial membrane antigen; H&E: Hamatoxylin and Eosin.

## Competing interests

In the past five years have you received reimbursements, fees, funding, or salary from an organization that may in any way gain or lose financially from the publication of this manuscript, either now or in the future? Is such an organization financing this manuscript (including the article-processing charge)? If so, please specify.

No

Do you hold any stocks or shares in an organization that may in any way gain or lose financially from the publication of this manuscript, either now or in the future? If so, please specify.

No

Do you hold or are you currently applying for any patents relating to the content of the manuscript? Have you received reimbursements, fees, funding, or salary from an organization that holds or has applied for patents relating to the content of the manuscript? If so, please specify.

No

Do you have any other financial competing interests? If so, please specify.

No

Non-financial competing interests.

No

Are there any non-financial competing interests (political, personal, religious, ideological, academic, intellectual, commercial or any other) to declare in relation to this manuscript? If so, please specify.

No

## Authors’ contributions

AMS: carried out the macroscopic handling, histological examination and immunohistochemical analysis of the lesion, reviewed the literature for histological differentials/mimics, took the microscopic photos and drafted the manuscript. PT: provided the clinical data, consented patient, provided the macroscopic photographs of the lesion and helped to draft the manuscript. WM: provided the soft tissue expertise into the diagnosis and differential diagnosis of the lesion, advised on the immunohistochemical panel and interpretation and contributed to the writing up. All authors read and approved the final manuscript.

## References

[B1] Nowak MA Marzich CS Scheetz KL McElroy JB Benign fibroepithelial polyps of the renal pelvis Arch Pathol Lab Med 1999 123 9 850 852 10458839 10.5858/1999-123-0850-BFPOTR

[B2] McCluggage WG A review and update of morphologically bland vulvovaginal mesenchymal lesions Int J Gynecol Pathol 2005 24 1 26 38 15626915

[B3] Ostor AG Fortune DW Riley CB Fibroepithelial polyps with atypical stromal cells (pseudosarcoma botryoides) of vulva and vagina. A report of 13 cases Int J Gynecol Pathol 1988 7 4 351 360 10.1097/00004347-198812000-00006 3229895

[B4] Nielsen GP Young RH Mesenchymal tumors and tumor-like lesions of the female genital tract: a selective review with emphasis on recently described entities Int J Gynecol Pathol 2001 20 2 105 127 10.1097/00004347-200104000-00002 11293156

[B5] Belli AK Somuncu E Aydogan T Bakkaloglu D Ilvan S Aydogan F Fibroepithelial polyp of the nipple in a woman Breast J 2013 19 1 111 112 10.1111/tbj.12063 23241056

[B6] Piubello Q Parisi A Eccher A Barbazeni G Franchini Z Iannucci A Flat epithelial atypia on core needle biopsy: which is the right management? Am J Surg Pathol 2009 33 7 1078 1084 10.1097/PAS.0b013e31819d0a4d 19390424

[B7] Souza LB Andrade ES Miguel MC Freitas RA Pinto LP Origin of stellate giant cells in oral fibrous lesions determined by immunohistochemical expression of vimentin, HHF-35, CD68 and factor XIIIa Pathology 2004 36 4 316 320 10.1080/00313020410001721627 15370129

[B8] Lucas EW Jr Branton P Mecklenburg FE Moawad GN Ectopic breast fibroadenoma of the vulva Obstet Gynecol 2009 114 2 Pt 2 460 462 19622961 10.1097/AOG.0b013e3181af672d

[B9] Kazakov DV Spagnolo DV Kacerovska D Michal M Lesions of anogenital mammary-like glands: an update Adv Anat Pathol 2011 18 1 1 28 10.1097/PAP.0b013e318202eba5 21169735

[B10] Brogi E Benign and malignant spindle cell lesions of the breast Semin Diagn Pathol 2004 21 1 57 64 10.1053/j.semdp.2003.10.007 15074560

[B11] El-Sayed ME Rakha EA Reed J Lee AH Evans AJ Ellis IO Predictive value of needle core biopsy diagnoses of lesions of uncertain malignant potential (B3) in abnormalities detected by mammographic screening Histopathology 2008 53 6 650 657 10.1111/j.1365-2559.2008.03158.x 19076681

[B12] Behtash N Mousavi A Tehranian A Khanafshar N Hanjani P Embryonal rhabdomyosarcoma of the uterine cervix: case report and review of the literature Gynecol Oncol 2003 91 2 452 455 10.1016/S0090-8258(03)00539-0 14599884

